# Enzyme adsorption-induced activity changes: a quantitative study on TiO_2_ model agglomerates

**DOI:** 10.1186/s12951-017-0283-4

**Published:** 2017-07-21

**Authors:** Augusto Márquez, Krisztina Kocsis, Gregor Zickler, Gilles R. Bourret, Andrea Feinle, Nicola Hüsing, Martin Himly, Albert Duschl, Thomas Berger, Oliver Diwald

**Affiliations:** 1 Department of Chemistry and Physics of Materials, Paris Lodron University of Salzburg, Jakob-Haringer-Strasse 2a, 5020 Salzburg, Austria; 2 Department of Molecular Biology, Paris Lodron University of Salzburg, Hellbrunnerstrasse 34/III, 5020 Salzburg, Austria

**Keywords:** Nanoparticles, Agglomerates, TiO_2_, β-galactosidase, Adsorption, Enzymatic activity, IR spectroscopy

## Abstract

**Background:**

Activity retention upon enzyme adsorption on inorganic nanostructures depends on different system parameters such as structure and composition of the support, composition of the medium as well as enzyme loading. Qualitative and quantitative characterization work, which aims at an elucidation of the microscopic details governing enzymatic activity, requires well-defined model systems.

**Results:**

Vapor phase-grown and thermally processed anatase TiO_2_ nanoparticle powders were transformed into aqueous particle dispersions and characterized by dynamic light scattering and laser Doppler electrophoresis. Addition of β-galactosidase (β-gal) to these dispersions leads to complete enzyme adsorption and the generation of β-gal/TiO_2_ heteroaggregates. For low enzyme loadings (~4% of the theoretical monolayer coverage) we observed a dramatic activity loss in enzymatic activity by a factor of 60–100 in comparison to that of the free enzyme in solution. Parallel ATR-IR-spectroscopic characterization of β-gal/TiO_2_ heteroaggregates reveals an adsorption-induced decrease of the β-sheet content and the formation of random structures leading to the deterioration of the active site.

**Conclusions:**

The study underlines that robust qualitative and quantitative statements about enzyme adsorption and activity retention require the use of model systems such as anatase TiO_2_ nanoparticle agglomerates featuring well-defined structural and compositional properties.

**Electronic supplementary material:**

The online version of this article (doi:10.1186/s12951-017-0283-4) contains supplementary material, which is available to authorized users.

## Background

A huge variety of synthetic approaches towards mesoporous materials with well-defined and tunable pore structures has become available only recently [1]. This has provided a promising new field to perform structure–activity studies at the microscopic level and for the development of novel hybrid materials for biocatalysis [2, 3] or biosensing [4, 5]. The functional properties of such materials are determined by the physicochemical parameters characterizing the interaction of biomolecules with the porous inorganic structure. The knowledge-based manipulation and optimization of these interactions requires well-defined mesoporous structures. At the same time, related model systems are also needed for insights into the interaction between biomolecules and engineered nanomaterials [6]. This applies for less defined and more dynamic structures such as particle agglomerates and/or aggregates, which are ubiquitous both in nature and in technology and which play an important role in emerging fields such as nanotoxicology or nanomedicine. Particle agglomerates and aggregates constitute complex structures and typically experience transformations in response to minute changes of the surrounding environment. For this reason, it is extremely difficult to establish model systems that mimic such a behavior sufficiently.

The properties of colloidal inorganic nanoparticles in biological media are subject to the adsorption of biomolecules and the formation of a protein corona around the particles [7, 8]. The generation of protein/particle composites affects the structure both of the protein (by adsorption-induced changes in protein conformation) and of the particles (by changes of the agglomeration and dispersion state) [9]. Since protein- and particle-related changes are strongly sensitive to a variety of chemical, physical and biological factors governing the interaction of the biomolecules with the inorganic material, enormous efforts have been devoted to the elucidation of related microscopic details [10–15]. Also for this purpose more systematic and quantitative studies involving reference systems are needed [13].

Vapor phase-grown particle powders of isolated anatase TiO_2_ nanocrystals and decontaminated particle surfaces have proven to be an appropriate model system for the study of hydration-induced microstructural and electronic property changes [16–20]. Only recently, we have employed such particle powders as model systems for protein adsorption studies and attained a reproducible qualitative and quantitative assessment of the interaction of a model serum protein (bovine serum albumin, BSA) with compositionally and structurally well-defined particle agglomerates. Such nanoparticle agglomerates are used in the present study to evaluate the impact of enzyme adsorption on its catalytic activity. For this purpose we selected β-galactosidase (β-gal) which has been used in previous model studies on enzyme immobilization at metal oxide nanomaterials [21–26]. Here we report the first qualitative and quantitative results obtained for enzyme adsorption using FT-IR spectroscopy and light scattering techniques and discuss challenges and pitfalls which typically arise during the evaluation of enzymatic activity changes between free and adsorbed proteins.

## Methods

### Chemicals

Na_2_CO_3_ (Sigma Aldrich, ACS reagent, ≥99.5%), Na_2_HPO_4_ (Sigma Aldrich, ACS Reagent ≥99%), citric acid (Sigma Aldrich, ≥99.5%), o-Nitrophenyl-β-D-galactopyranoside (ONPG, Sigma Aldrich, >99% HPLC), MgCl_2_ (Sigma Aldrich, ACS Reagent >99%) and D_2_O (Aldrich, 99 atom% D) were used as received. β-gal was produced in house as a highly purified recombinant protein according to protocols previously described in detail [27]. All H_2_O solutions were prepared using water with a resistivity of 18 M Ω cm (Millipore, Milli-Q).

### Nanoparticle synthesis

Anatase TiO_2_ nanocrystals were prepared by metal organic chemical vapor synthesis (MOCVS) based on the decomposition of titanium (IV) isopropoxide at *T* = 1073 K in a hot wall reactor system [28, 29]. For purification, the obtained powder samples were subjected to thermal treatment under high vacuum conditions (*p* < 10^−5^ mbar). First, the powder sample was heated to *T* = 873 K using a rate of *r* ≤ 5 K min^−1^. Subsequent oxidation with O_2_ at this temperature was applied to remove organic remnants from the precursor material and to guarantee the stoichiometric composition of the oxide. Such post-synthesis treatment eliminates organic remnants as evidenced by IR spectroscopy [29]. With transmission electron microscopy (TEM) we found that the majority of crystallites display a highly irregular but approximately equidimensional shape (Additional file 1: Figure S1) [28, 29]. The average diameter of these nanocrystals corresponds to 13 nm. The specific surface area deduced from the TEM mean diameter value corresponds to 121 m^2^ g^−1^ and is in good agreement with results from nitrogen sorption measurements (130 ± 13 m^2^ g^−1^) [28].

### Preparation and characterization of particle dispersions

The size distribution and zeta potential of agglomerates was determined for aqueous TiO_2_ particle dispersions with a concentration of 0.1 mg mL^−1^. To ensure sufficient particle dispersion an ultrasonic finger (amplitude 25%, 30 min, UP200St, Ti-sonotrode 2 mm, Hielscher Ultrasonics GmbH) was used while the dispersion was cooled in an ice bath to prevent heating via mechanical sample agitation. Protein adsorption was performed at room temperature by mixing 15 mL of TiO_2_ particle dispersion with 5 mL of protein solution (final particle concentration: [TiO_2_] = 0.1 mg mL^−1^, [β-gal] = 50 µg mL^−1^). The dispersion was then mechanically stirred for 12 h.

We will refer to secondary particles, which are built up from TiO_2_ nanocrystals in enzyme-free water, as agglomerates. Nanocrystals in agglomerates are held together by weak physical interactions. On the other hand we refer to β-gal/TiO_2_ composites forming after the addition of enzyme to preformed agglomerates as heteroaggregates. Protein adsorption onto the oxide is expected to involve not only weak physical interactions, but also chemical bonding.

A Zetasizer Nano ZSP ZEN5600 (Malvern Instruments) was used to determine the size distribution of dispersed TiO_2_ particles, proteins and protein/TiO_2_ heteroaggregates by dynamic light scattering (DLS) as well as the zeta potentials by laser Doppler electrophoresis. To obtain size distribution functions from DLS measurements 70 size classes were used for TiO_2_ dispersions and 300 size classes for enzyme solutions. To transform the intensity distribution of hydrodynamic diameters into a number distribution several assumptions have to be made, including that all particles are spherical and homogeneous. To support data obtained from DLS we therefore performed an additional electron microscopic study of TiO_2_ agglomerates and β-gal/TiO_2_ heteroaggregates.

### Electron microscopic characterization of TiO_2_ agglomerates and β-gal/TiO_2_ heteroaggregates

After DLS analysis small volumes of aqueous dispersions containing TiO_2_ agglomerates or β-gal/TiO_2_ heteroaggregates were dropped onto a carbon film on a Cu grid. The immobilized samples were then dried at room temperature. The samples were imaged with a field emission gun SEM Ultra Plus 55 (Zeiss) at an accelerating voltage of 5 kV with the Gemini In-Lens secondary electron detector. For related TEM measurements a TECHNAI F20 microscope equipped with a field emission gun and S-twin objective lens was used.

### Activity of free and immobilized β-gal-enzymatic assay

The activity of β-gal was studied at 5.5 < pH < 8.5 in McIlvaine’s buffer (0.1 M citric acid and 0.2 M Na_2_HPO_4_ at different ratios) both in the free state ([β-gal] = 0.5 µg mL^−1^) and when adsorbed on TiO_2_ nanoparticle agglomerates ([TiO_2_] = 1 mg mL^−1^, [β-gal] = 8.5 µg mL^−1^). MgCl_2_ (1 mM) and ONPG (0.5 mM) were used as cofactor and substrate, respectively.

An aqueous TiO_2_ particle dispersion was first treated ultrasonically and then mixed with aqueous β-gal solution. The dispersion was mechanically stirred (VWR VMS-C4, 600 rpm) at room temperature for 12 h. Finally the sample was centrifuged (EBA 20 centrifuge, Hettich, 6000 rpm). Bradford analysis confirmed that enzyme adsorption was complete and leads to complete enzyme elimination from the supernatant. The β-gal/TiO_2_ heteroaggregates were washed first with water and then with the respective buffer solution. Finally, the enzymatic reaction was started by adding ONPG to the buffer and agitation of the mixture. The reaction progress was monitored as a function of time by withdrawing 0.5 mL of the reaction mixture and mixing with 0.7 mL of 1 M Na_2_CO_3_ aqueous solution to stop the reaction. The resulting dispersion was centrifuged (Biofuge fresco, Heraeus, 13,000 rpm) and the concentration of the reaction product (o-nitrophenol, ONP) was determined photometrically at a wavelength *λ* = 420 nm (molar extinction coefficient, *ε* (420 nm) = 2.13·10^4^ M^−1^ cm^−1^ at pH 10.2) [30]. The specific enzyme activity is represented in U/mg, where one enzyme unit (U) corresponds to the amount of enzyme catalysing the reaction of 1 µmol of ONP per minute.

### Study of protein conformation—ATR-FTIR-spectroscopy

For IR measurements an attenuated total reflection (ATR) unit (PIKE Technologies, Veemax II) was attached to a Bruker Vertex 70 FTIR spectrometer equipped with a MCT detector. The measurements were performed at an incident angle of 55° using a hemispherical ZnSe prism. Spectra were obtained by averaging 100 scans at a resolution of 4 cm^−1^ and are represented as –log (R/R_0_), where R and R_0_ are the reflectance values corresponding to the single beam spectra recorded for sample and reference, respectively.

#### Proteins in solution

First, the protein solution was applied to the ATR prism and spectra were recorded until the establishment of adsorption equilibrium. Afterwards, the prism was rinsed several times with pure water. A spectrum of the hydrated protein layer which remains at the prism surface serves as the reference. Finally, the pure water was replaced again by the respective protein solution and the sample spectrum was measured. This procedure assures that the detected signals result exclusively from proteins in solution and not from the protein layer at the surface of the ATR prism.

#### Proteins adsorbed on a porous TiO_2_ nanoparticle film

A TiO_2_ film was immobilized on the ATR prism by doctor blade deposition using Scotch tape as spacer. First, 40 µL of a 0.4 M TiO_2_ nanoparticle dispersion was applied per cm^2^ of prism surface and spread over it. The resulting film was then dried in a nitrogen stream. Afterwards, the IR cell was assembled by pressing a glass cell against the pre-coated prism using a Teflon ring as the junction. Finally, the cell was filled with water or aqueous protein solution to measure the background or sample spectrum, respectively.

#### Spectrum fitting

The amide I and II region of protein spectra (1700–1480 cm^−1^) was fitted by a set of Gaussian-shaped bands. From the second derivative of an experimental spectrum we determined the minima and defined related values as band positions [31, 32]. Prior to spectrum fitting a horizontal baseline, which was extrapolated from the flat spectral region between 2000 and 1800 cm^−1^, was subtracted from the original spectrum. All band positions and band widths were kept constant regardless of whether the protein was free in aqueous (H_2_O or D_2_O) solution or adsorbed on TiO_2_. Spectra were fitted by optimizing the contribution of single bands using iterative Chi square minimization (Levenberg–Marquardt algorithm). For visualization of the protein structure of β-gal the UCSF Chimera software tool [33] was used.

## Results and discussion

### β-gal adsorption-induced colloidal property changes of vapor phase-grown TiO_2_ nanoparticles in aqueous dispersion

**Fig. 1:**
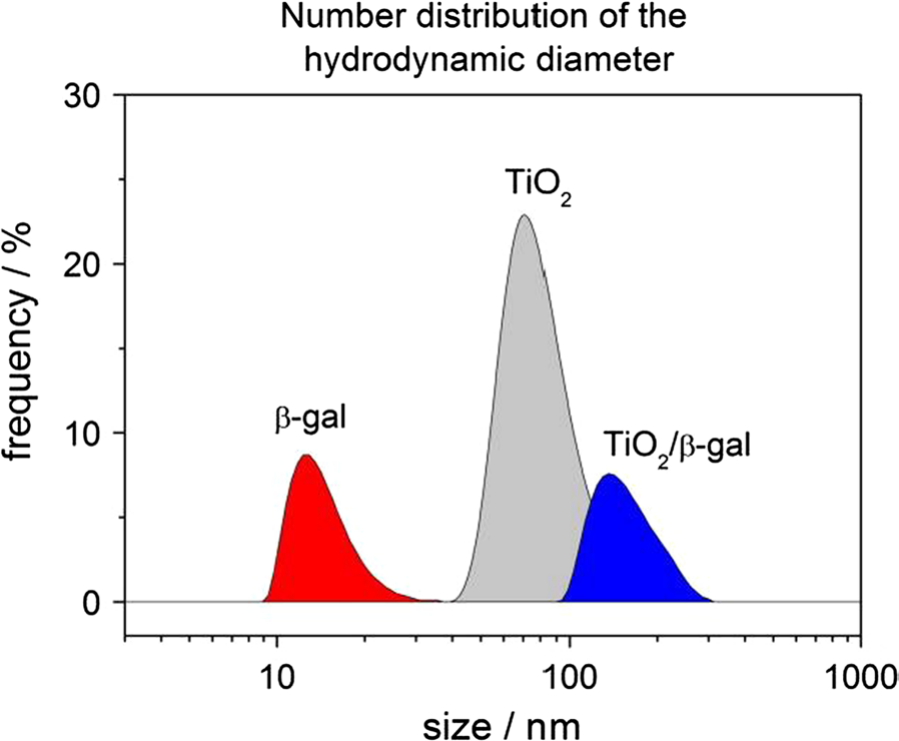
Particle size distribution as related to β-gal, TiO_2_ agglomerates and mixtures of β-gal and TiO_2_ agglomerates in aqueous suspension. A size increment from ~80 to ~140 nm reveals the adsorption of β-gal and the formation of β-gal/TiO_2_ composites. [β-gal] = 50 µg mL^−1^; [TiO_2_] = 0.1 mg mL^−1^

Anatase TiO_2_ nanoparticle powders, which were grown in the gas phase and processed in vacuum, are characterized by ensembles of equidimensional nanoparticles (primary particle size: 13 nm) with low concentrations of solid–solid interfaces and contaminant-free surfaces (Additional file 1: Figure S1) [16]. After ultrasonic treatment particles form agglomerates in aqueous dispersion, which display agglomerate size distributions peaking at ~80 nm (Fig. 1). The hydrodynamic diameter of β-gal in aqueous solution was determined to be ~13 nm (Fig. 1). As tracked by DLS, β-gal adsorption onto TiO_2_ agglomerates increases the agglomerate size. Final particle size distributions with a maximum at ~140 nm (Fig. 1) suggest the formation of β-gal/TiO_2_ heteroaggregates, where an enzyme corona forms at the outer surface of TiO_2_ agglomerates. TiO_2_ de-agglomeration by enzyme adsorption followed by a re-agglomeration upon the formation of larger β-gal/TiO_2_ heteroaggregates represents an alternative scenario. However, only the protein free dispersions of vapor-phase grown TiO_2_ particles were subjected to an ultrasonic treatment in order to achieve model agglomerates with reproducible particle size distribution. After enzyme addition, we minimized further input of mechanical energy and stirred the dispersions only mildly until an adsorption equilibrium was reached. We expect that further breakup of the TiO_2_ agglomerates and subsequent protein inclusion during re-agglomeration is rather unlikely under such conditions. Adsorption-induced changes of the agglomerates’ surface charge provide strong evidence for protein corona formation, i.e. the generation of a ß-gal shell around TiO_2_ agglomerates. The zeta potential of TiO_2_ agglomerates in aqueous suspension decreases from its starting value related to pure TiO_2_ dispersions (−3.0 mV) to that of β-gal—containing dispersions (−27 mV) corresponding to the zeta potential of β-gal in solution (−29 mV).

**Fig. 2:**
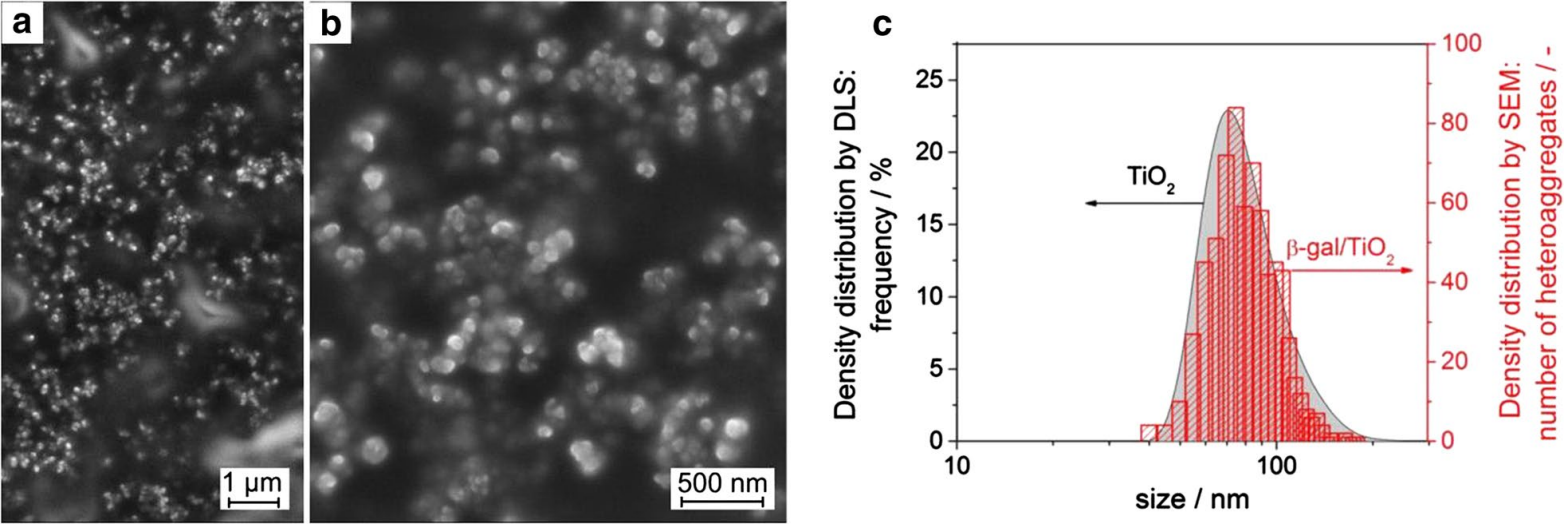
**a**, **b** Scanning electron micrographs of β-gal/TiO_2_ heteroaggregates. **c** Number-weighted size distribution of β-gal/TiO_2_ heteroaggregates as determined from the electron micrographs (**a**, **b**) and of TiO_2_ agglomerates as determined by DLS. Samples for SEM analysis were prepared from dispersions previously analyzed by DLS (Fig. 1)

To complement data obtained from DLS we performed an electron microscopic study of TiO_2_ agglomerates and β-gal/TiO_2_ heteroaggregates. For this purpose we used conventional scanning electron microscopy (SEM, Fig. 2) and transmission electron microscopy (TEM, Additional file 1: Figure S1). It has to be emphasized that the immobilization of colloidal particles onto a sample grid is associated with a change of the particle surrounding from a condensed liquid phase to a high vacuum environment. It is well known that such a transformation may be associated with a significant modification of the microstructure. Furthermore, care has to be taken when deducing from a limited number of microscopically resolved and analyzed entities generalized conclusions about the structural properties of a large particle ensemble. Indeed we observe on the same sample grid different microstructures. On the one hand, extended almost two-dimensional structures are observed, which can conveniently be studied by TEM (Additional file 1: Figure S1). We tentatively associate the formation of these structures with a drying-induced spreading of primary particles on the substrate both for TiO_2_ agglomerates and for β-gal/TiO_2_ heteroaggregates. Importantly, the primary particle size does not change upon enzyme adsorption (Additional file 1: Figure S1). On the other hand, we observe sample spots characterized by highly agglomerated or aggregated secondary particles, which feature however a quite narrow size distribution. The resulting structures seem to be three-dimensional thus escaping TEM investigation. However, from scanning electron micrographs we determined the number-weighted size distribution of β-gal/TiO_2_ heteroaggregates (Fig. 2). Remarkably, the size distribution of β-gal/TiO_2_ heteroaggregates resembles quite closely the size distribution of TiO_2_ agglomerates in dispersion. Whereas the adsorbed protein layer escapes detection by SEM, these results suggest that TiO_2_ agglomerates serve as substrates for enzyme adsorption upon conservation of their original size. On the other hand, this observation suggests that a high local concentration of agglomerates is beneficial for avoiding drying artefacts upon sample preparation thus preserving the original secondary particle size in dispersion. At the moment, however, we do not fully control the parameters governing sample deposition on the microscopy grid. Further work in this direction is underway.

To estimate the number of β-gal molecules being adsorbed per TiO_2_ agglomerate we used the Bradford assay. The supernatant solution confirms complete enzyme adsorption. Moreover, we neglected enzyme adsorption at the walls of the glass beaker and considered its adsorption on TiO_2_ agglomerates with a size of 80 nm and a solid fraction of 0.5 (i.e. 50% porosity) [16]. On this basis we estimated the number of 325 β-gal molecules per agglomerate. A rough estimate^1^ of the number of β-gal molecules which would correspond to a protein monolayer covering the external surface of TiO_2_ agglomerates yields ~150 β-gal molecules per TiO_2_ agglomerate. A comparison with the experimental value (325 β-gal molecules per TiO_2_ agglomerate) led us to the conclusion that β-gal may form multilayers at the outer surface of TiO_2_ agglomerates. This is different to BSA adsorption on comparable TiO_2_ agglomerate structures where we observed saturation at the level of one monolayer equivalent as reported only recently [34].

### Adsorption of β-gal and the impact on enzymatic activity

β-gal (molecular weight: 465,000 g mol^−1^) hydrolyzes lactose and other β-galactosides into monosaccharides. For the following experiments we used heteroaggregated β-gal/TiO_2_ systems and explored how enzyme adsorption on TiO_2_ agglomerates impacts on the enzyme’s biological activity. More specifically, we focus on the interaction of isolated enzyme molecules with the inorganic substrate. To exclude significant protein–protein interactions such as protein aggregation and multilayer formation at the oxide surface, agglomerate coverage was kept below one monolayer equivalent.

After an adsorption time of 12 h, Bradford analysis clearly reveals that the enzyme ([β-gal] = 8.5 µg mL^−1^) was completely removed from the aqueous solution by the dispersed TiO_2_ agglomerates ([TiO_2_] = 1 mg mL^−1^). This uptake corresponds to ~6 β-gal molecules per TiO_2_ agglomerate, or ~4% of a theoretical monolayer. The β-gal/TiO_2_ heteroaggregates were first washed with water and then treated in the respective buffer solution.

**Fig. 3:**
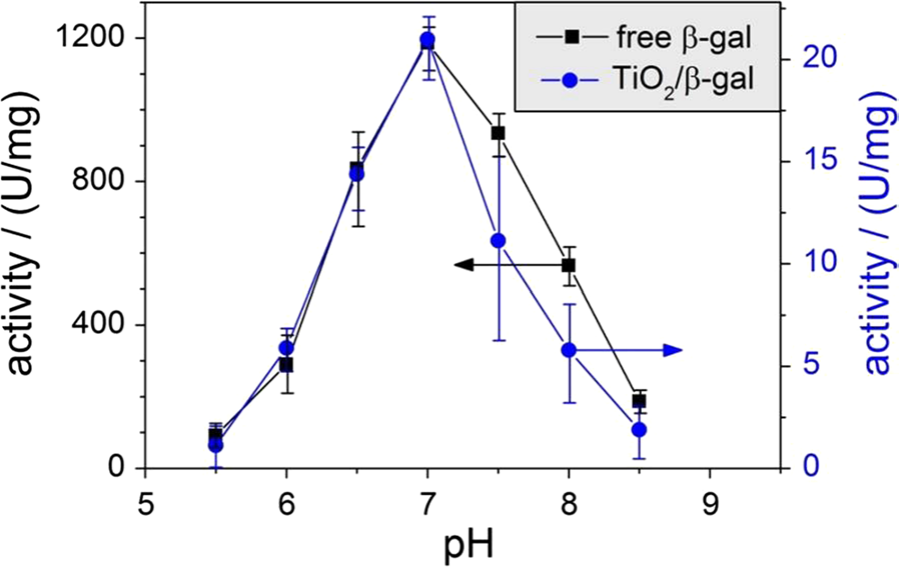
Enzymatic activity of β-gal in McIlvaine’s buffer both in the free state ([β-gal] = 0.5 µg mL^−1^) and adsorbed on TiO_2_ nanoparticle agglomerates ([TiO_2_] = 1 mg mL^−1^, [β-gal] = 8.5 µg mL^−1^). Ordinate scales on the *left* and the *right* hand side correspond to the activities of free and adsorbed β-gal, respectively, as indicated by *arrows*

In comparison to the protein in free form ([β-gal] = 0.5 µg mL^−1^) the enzymatic activity of the protein adsorbed on TiO_2_ agglomerates (Fig. 3; Additional file 1: Figure S2) was studied in the range 5.5 < pH < 8.5 using a McIlvaine’s buffer (0.1 M citric acid and 0.2 M Na_2_HPO_4_ at different ratios). Consistent with previous studies [26] the activity of free β-gal was observed to peak at ~pH 7. Upon adsorption the enzymatic activity decreases drastically by a factor of 60 at pH ≤ 7. At pH 6.5 the activity of adsorbed β-gal was also tested in pure water (i.e. in the absence of the buffer) yielding a comparable activity. For the regime pH > 7 we observed an even more pronounced decrease—by a factor of ~100—of the apparent enzyme activity.

**Fig. 4:**
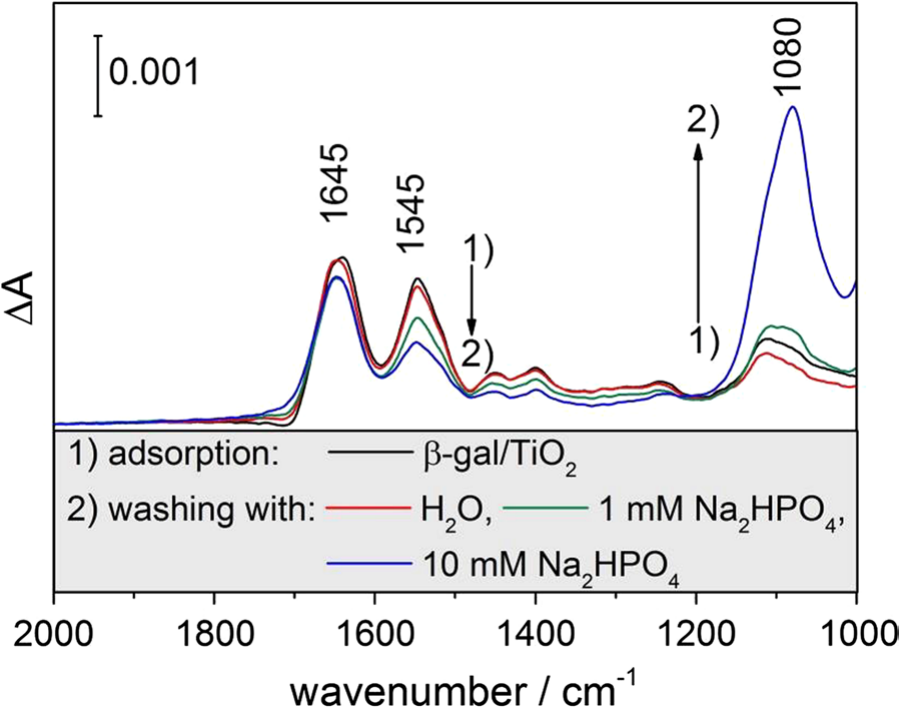
ATR-IR spectra of β-gal adsorbed on a porous film of TiO_2_ nanoparticles ([β-gal] = 150 µg mL^−1^, [TiO_2_] = 1.3 mg mL^−1^, adsorption time: 8 h), background spectrum: TiO_2_ film in contact with water. Following protein adsorption the film was extensively rinsed first with water and then with Na_2_HPO_4_ solutions ([Na_2_HPO_4_] = 1 and 10 mM, respectively)

Buffer species in solution are needed for the stabilization of the pH during the activity measurements. Their potential competition with proteins for adsorption sites needs to be included in the present discussion. ${\text{HPO}}_{4}^{{2 - }}$ bind as bidentate species and—in principle—can also induce protein desorption [35]. At this point, we hypothesize that the observed deviation of the activity trend (Fig. 3) above pH > 7 is due to buffer-induced desorption of β-gal, since at pH values between 7.2 and $8.5\;{\text{HPO}}_{4}^{{2 - }}$ is the most abundant phosphate species (H_3_PO_4_: pK_a1_ = 2.15, pK_a2_ = 7.2, pK_a3_ = 12.4). To test this hypothesis we studied the effect of ${\text{HPO}}_{4}^{{2 - }}$ on the concentration of adsorbed β-gal using ATR-IR spectroscopy on a porous film of vapor phase grown TiO_2_ nanoparticles (Fig. 4).^2^ Upon β-gal adsorption protein-specific amide bands at 1645 (amide I) and 1545 cm^−1^ (amide II) appear in the IR spectrum, the intensities of which remain constant after removal of the protein solution and extensive washing with pure H_2_O. However, washing the film with Na_2_HPO_4_ solutions of increasing concentration clearly depletes the intensities of the protein-specific bands and generates a new band at 1080 cm^−1^ characteristic of ${\text{HPO}}_{4}^{{2 - }}$ [36]. Buffer induced protein desorption was also observed when using HEPES (4-(2-hydroxyethyl)-1-piperazineethanesulfonic acid) instead of McIlvaine’s buffer (not shown).

Several aspects have to be considered when comparing the activity of enzymes in free and adsorbed form. First of all, the number of enzyme molecules involved has to be known. While this type of information can be obtained in a straightforward manner for proteins in solution, related efforts are challenging with regard to adsorbed proteins. The total number of adsorbed molecules may change significantly upon minute changes of solution composition as shown above (Fig. 4). Furthermore, mass transport and, thus, local gradients of reaction educts and products are significantly influenced by (a) the distribution of the enzyme within pores or at the oxide surface (sub-monolayer, monolayer, multilayers) and (b) the distribution of enzyme/oxide heteroaggregates within the reaction volume (i.e. their dispersion state). The dispersion state in turn depends on protein coverage and solution composition and pH.

Finally, the protein/particle ratio and, more precisely, the coverage of the surface available on single particles, agglomerates or aggregates need to be specified. For instance, it has been shown that the degree of conformational change of adsorbed proteins depends on the protein/particle surface ratio [37]. In the following we use ATR-IR spectroscopy to probe the structural changes β-gal experiences during adsorption.

Experiments on the formation of the protein corona focus for individual proteins often on the formation of a complete protein shell where the entire particle surface is covered [38, 39]. In the present study, only 4% of the surface was covered with protein, which is more realistic for any individual protein when nanoparticles have entered the human body. Of note, buffering compounds will be present in such a situation.

### Structural changes of β-gal adsorbed on porous films of vapor phase-grown TiO_2_ nanoparticles

The ATR-IR spectra of β-gal adsorbed from aqueous solution on porous films of vapor phase-grown TiO_2_ nanoparticles (Fig. 4; Additional file 1: Figure S3) are characterized by two characteristic protein-specific bands at 1645 cm^−1^ (amide I) and 1545 cm^−1^ (amide II) [31, 32, 40, 41]. A detailed analysis of ATR-IR spectra was performed to explore adsorption-induced changes in protein conformation. The amide I band intensity exhibits sensitive dependence on the protein backbone, since different secondary structures contribute to the amide I band in a narrow wavenumber range [31, 32, 42]. β-gal is a tetramer, which is mainly composed of α-helical and β-sheet segments and which has smaller contributions from random coil and β-turns [43]. Every tetramer contains four functional active sites. The active site is formed primarily by an α/β barrel structure of each monomer, however, it includes also critical catalytic residues of other monomers [44].

**Fig. 5:**
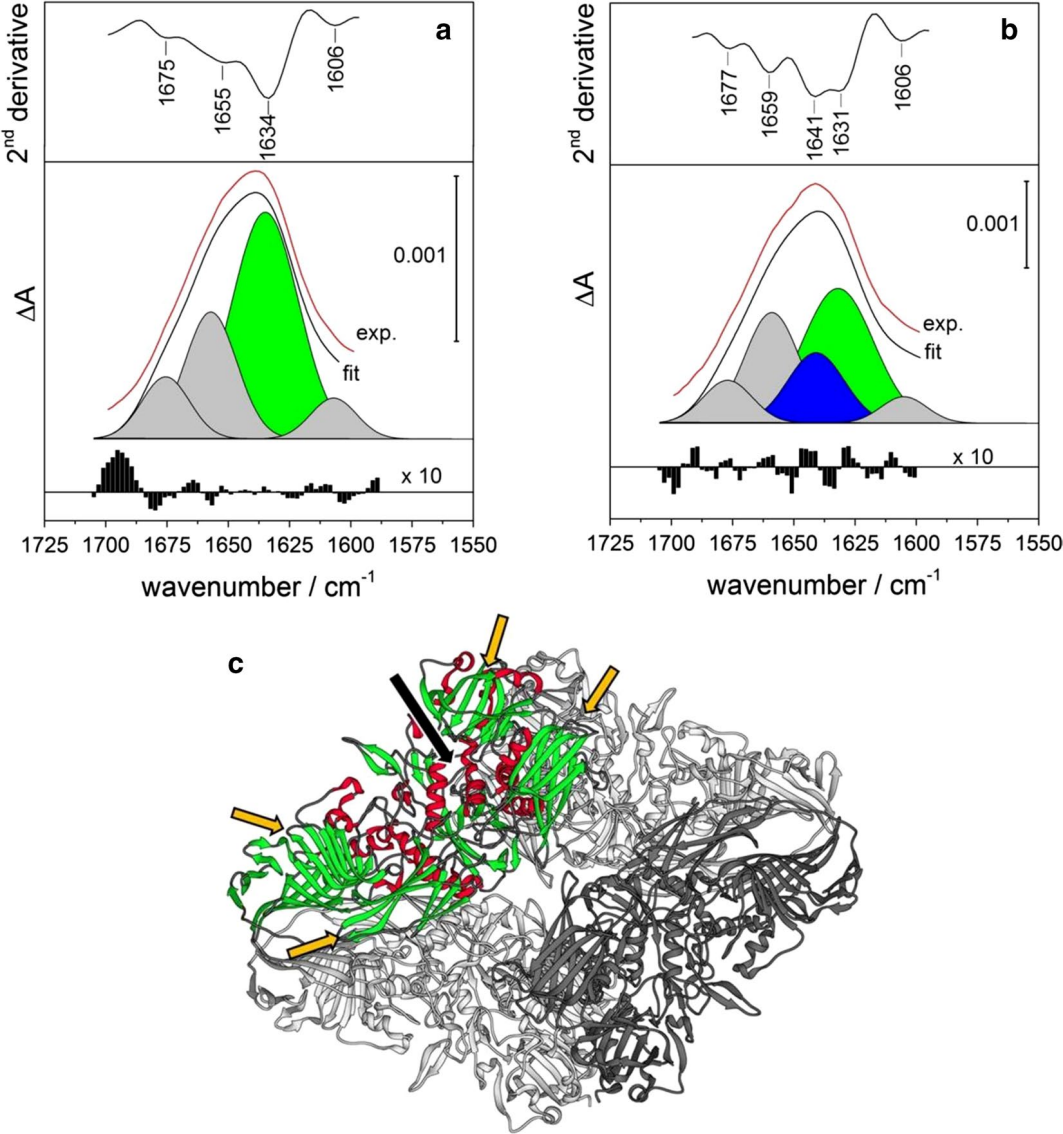
Second derivative of the amide I band, fitting results and corresponding residuals for **a** free β-gal in D_2_O ([β-gal] = 150 µg mL^−1^) and **b** β-gal adsorbed on a porous film of TiO_2_ nanoparticles ([β-gal] = 150 µg mL^−1^, [TiO_2_] = 1.3 mg mL^−1^, adsorption time: 8 h), background spectrum: TiO_2_ film in contact with D_2_O. The band parameters of the deconvoluted single components are listed in Table 1. **c** Graphical representation of the protein structure of β-gal retrieved from the Protein Databank (pdb entry: 1f4a) [45] with the α/β barrel carrying the active center of one of the four monomers (indicated by a *black arrow*) surrounded by four domains rich in β-sheet structures (marked by *yellow arrows*). α-helices and β-sheets of one monomer are highlighted in *red* and *green*, respectively

IR spectra of β-gal both in aqueous (H_2_O or D_2_O) solution and adsorbed on a TiO_2_ film are shown in the Additional file 1: Figure S3. The spectral range between 1700 and 1480 cm^−1^ which features the amide I and amide II bands (Additional file 1: Figures S4, S5) was subjected to a band deconvolution procedure. Additional file 1: Figure S6 shows the fitting result for the amide I band of β-gal (free and adsorbed) in an H_2_O environment. Upon adsorption a significant decrease of the component at ~1630 cm^−1^ (attributed to β-sheet structures) and an increase of the component at ~1655 cm^−1^ (attributed to α-helix and random structures) is observed (Additional file 1: Figure S6, Table S1). With regard to the fitting of the amide I band the overlap of α-helix and random structures represents a fundamental problem in H_2_O. This overlap can be greatly reduced in D_2_O due to small band shifts of the amide I components upon a hydrogen/deuterium exchange [31, 32]. Furthermore, interference from the H_2_O bending mode, which overlaps with the amide I band, can be eliminated in D_2_O. The experimental IR spectrum and the corresponding fitting results for β-gal (free and adsorbed) in a D_2_O environment are provided in Fig. 5 and in the Additional file 1: Figures S3 and S5. The second derivative of the spectrum corresponding to adsorbed β-gal (Fig. 5b) features a contribution at 1641 cm^−1^, which is absent in the spectrum of the free protein (Fig. 5a) and which is attributed to random structures. From the fitting results it can be deduced that this contribution grows on the expense of the β-sheet content, which is reduced by ~30% as compared to the free protein, while the structure of the α-helices remains essentially undisturbed. The adsorption induced deterioration of β-sheets is intimately related to the structure of β-gal: [44] the central domain forming the α/β barrel structure (indicated by the black arrow in Fig. 5c) constitutes the core of each monomer and is surrounded by four domains containing mainly β-sheet structures (indicated by yellow arrows in Fig. 5c). These outer parts of the protein preferentially interact with the oxide surface upon adsorption. The associated conversion of β-sheets into random structures and a modification of the active sites are expected to significantly contribute to the dramatic decrease of the enzymatic activity upon adsorption. The restricted accessibility of reactive centers upon enzyme adsorption may additionally contribute to the observed activity loss (Table 1).

**Table 1:** Band parameters of deconvoluted single components contributing to the amide I band of free β-gal in D_2_O and β-gal adsorbed on TiO_2_ (corresponding to the fitting results represented in Fig. 5)

Structure	Free β-gal			Adsorbed β-gal		
	Peak position (cm^−1^)	FWHM (cm^−1^)	Area (%)	Peak position (cm^−1^)	FWHM (cm^−1^)	Area (%)
Inter β-sheet	1606	22	7	1606	22	5
β-Sheet	1634	32	58	1631	34	42
Random	–	–	–	1641	27	17
α-Helix	1655	25	25	1659	26	27
Turn	1675	23	10	1677	23	9

The results presented here describe a situation where the local environment of the enzyme is strongly influenced by the inorganic structure of the TiO_2_ agglomerates due to a high enzyme dispersion at the agglomerate surface. At low surface coverages, this leads to a significant modification of the protein conformation and, consequently, to a dramatic loss in enzyme activity. As the extent of conformational change of a protein adsorbed onto nanostructured materials may critically depend on coverage [37], the present situation corresponds to the worst case with regard to the conservation of the enzyme activity upon immobilization. Immobilization strategies which involve enzyme adsorption may involve enzyme distortion-related activity losses [46]. While at low enzyme loadings the contact area between the enzyme and the substrate may be maximized, contact area minimization does occur at higher loadings [46]. For low enzyme coverages the co-adsorption of proteins (e.g. BSA) or polymers has been found to improve the activity of enzymes immobilized on 2D or microstructured substrates [46, 47]. Similar effects have been reported for materials which are structured at lower length scales, i.e. for nanoparticulate systems [48].

The systematic investigation of coverage-dependent adsorption processes including associated protein conformation changes and, as a result therefrom, competitive processes require rigorous control of both the structural properties of nanoparticle agglomerates and of the enzyme loading. We believe that the characterization approach presented here, i.e. the combination of systematic and quantitative adsorption studies and molecular insight from spectroscopy, can only be performed on structurally and compositionally well-defined model agglomerates. Related reference systems may serve as a valuable tool for the future investigation of more complex bio/nano hybrid systems containing commercially available components.

## Conclusions

Enzymatic activity changes of β-gal when adsorbed at low coverages (~4% of the theoretical monolayer coverage) on compositionally well-defined anatase TiO_2_ agglomerates were linked to results from complementary FT-IR measurements. Substantial loss in biological activity was observed over the entire pH range investigated, i.e. 5.5 < pH < 8.5, and corresponds to a decrease by a factor of 60–100. Band analysis of the FT-IR active amide modes clearly revealed the adsorption-induced deterioration of β-sheet structures and—as a result—the origin of the deactivation of the catalytic site. The here presented combination between surface science-inspired and molecular spectroscopy based adsorption studies on oxide nanomaterials also include the determination of size and surface charge of the metal oxide-based agglomerates and requires well-defined particulate model systems with pre-processed and bare oxide surfaces. Robust qualitative and quantitative statements about enzyme adsorption-induced activity changes become accessible only on such systems. Moreover, they allow for insights into cooperative adsorption effects involving different types of proteins or other molecules from the buffer solution.

## Additional file

**Additional file 1.** Transmission electron micrographs, hydrolysis plots, IR spectra and fitting results.

## Abbreviations

ATR: attenuated total reflection
BSA: bovine serum albumin
DLS: dynamic light scattering
HEPES: 4-(2-attenuated)-1-piperazineethanesulfonic acid
MOCVS: metal organic chemical vapor synthesis
ONP: o-nitrophenol
ONPG: o-nitrophenyl-β-D-galactopyranoside
SEM: scanning electron microscopy
TEM: transmission electron microscopy
β-gal: β-galactosidase

## Footnotes

1 For this purpose, agglomerates were modeled by spheres with a diameter of 80 nm (i.e. the hydrodynamic diameter determined by DLS, Fig. 1). The required adsorption area per β-gal molecule was approximated by the projection area of a spherical molecule with a diameter of 13 nm (i.e. the hydrodynamic diameter of β-gal in solution, Fig. 1) to a planar surface. This approximation yields a monolayer coverage of 150 β-gal molecules per TiO_2_ agglomerate.

2 Due to sensitivity reasons we had to use for ATR-IR experiments a [β-gal]/[TiO_2_] ratio, which is about 14 times higher than the one used in the enzymatic assay. However, using as the basis for enzyme quantification the same considerations as in Footnote 1, this concentration corresponds to ~50% of a theoretical monolayer.

## References

[1] Feinle A, Elsässer MS, Hüsing N (2016). Sol–gel synthesis of monolithic materials with hierarchical porosity. Chem Soc Rev.

[2] Zhou Z, Hartmann M (2013). Progress in enzyme immobilization in ordered mesoporous materials and related applications. Chem Soc Rev.

[3] Fried DI, Brieler FJ, Fröba M (2013). Designing inorganic porous materials for enzyme adsorption and applications in biocatalysis. ChemCatChem.

[4] Bhakta SA, Evans E, Benavidez TE, Garcia CD (2015). Protein adsorption onto nanomaterials for the development of biosensors and analytical devices: a review. Anal Chim Acta.

[5] Topoglidis E, Cass A, O’Regan B, Durrant JR (2001). Immobilisation and bioelectrochemistry of proteins on nanoporous TiO_2_ and ZnO films. J Electroanal Chem.

[6] Monopoli MP, Pitek AS, Lynch I, Dawson KA (2013). Formation and characterization of the nanoparticle-protein corona. Methods Mol Biol.

[7] Lynch I, Cedervall T, Lundqvist M, Cabaleiro-Lago C, Linse S, Dawson KA (2007). The nanoparticle-protein complex as a biological entity; a complex fluids and surface science challenge for the 21st century. Adv Colloid Interf Sci.

[8] Vidic J, Haque F, Guigner JM, Vidy A, Chevalier C, Stankic S (2014). Effects of water and cell culture media on the physicochemical properties of ZnMgO nanoparticles and their toxicity toward mammalian cells. Langmuir.

[9] Schulze C, Kroll A, Lehr CM, Schäfer UF, Becker K, Schnekenburger J, Schulze Isfort C, Landsiedel R, Wohlleben W (2008). Not ready to use-overcoming pitfalls when dispersing nanoparticles in physiological media. Nanotoxicology.

[10] Nel AE, Mädler L, Velegol D, Xia T, Hoek E, Somasundaran P, Klaessig F, Castranova V, Thompson M (2009). Understanding biophysicochemical interactions at the nano-bio interface. Nat Mater.

[11] Mahmoudi M, Lynch I, Ejtehadi MR, Monopoli MP, Bombelli FB, Laurent S (2011). Protein-nanoparticle interactions: opportunities and challenges. Chem Rev.

[12] Walkey CD, Chan W (2012). Understanding and controlling the interaction of nanomaterials with proteins in a physiological environment. Chem Soc Rev.

[13] Pino PD, Pelaz B, Zhang Q, Maffre P, Nienhaus GU, Parak WJ (2014). Protein corona formation around nanoparticles-from the past to the future. Mater Horizons.

[14] Kumar A, Das S, Munusamy P, Self W, Baer DR, Sayle DC, Seal S (2014). Behavior of nanoceria in biologically-relevant environments. Environ Sci Nano.

[15] Mudunkotuwa IA, Grassian VH (2015). Biological and environmental media control oxide nanoparticle surface composition: the roles of biological components (proteins and amino acids), inorganic oxyanions and humic acid. Environ Sci Nano.

[16] Elser MJ, Berger T, Brandhuber D, Bernardi J, Diwald O, Knözinger E (2006). Particles coming together: electron centers in adjoined TiO_2_ nanocrystals. J Phys Chem B.

[17] Siedl N, Elser MJ, Halwax E, Bernardi J, Diwald O (2009). When fewer photons do more: a comparative O_2_ photoadsorption study on vapor-deposited TiO_2_ and ZrO_2_ nanocrystal ensembles. J Phys Chem C.

[18] Baumann SO, Elser MJ, Auer M, Bernardi J, Hüsing N, Diwald O (2011). Solid–solid interface formation in TiO_2_ nanoparticle networks. Langmuir.

[19] Elser MJ, Diwald O (2012). Facilitated lattice oxygen depletion in consolidated TiO_2_ nanocrystal ensembles: a quantitative spectroscopic O_2_ adsorption study. J Phys Chem C.

[20] Berger T, Diwald O, Jupille J, Thornton J (2015). Defects in metal oxide nanoparticle powders. Defects at oxide surfaces.

[21] Di Serio M, Maturo C, de Alteriis E, Parascandola P, Tesser R, Santacesaria E (2003). Lactose hydrolysis by immobilized β-galactosidase: the effect of the supports and the kinetics. Catal Today.

[22] Satyawali Y, Roy SV, Roevens A, Meynen V, Mullens S, Jochems P, Doyen W, Cauwenberghs L, Dejonghe W (2013). Characterization and analysis of the adsorption immobilization mechanism of β-galactosidase on metal oxide powders. RSC Adv.

[23] Bernal C, Sierra L, Mesa M (2011). Application of hierarchical porous silica with a stable large porosity for β-galactosidase immobilization. ChemCatChem.

[24] Bernal C, Sierra L, Mesa M (2012). Improvement of thermal stability of β-galactosidase from Bacillus circulans by multipoint covalent immobilization in hierarchical macro-mesoporous silica. J Mol Catal B.

[25] Verma ML, Barrow CJ, Kennedy JF, Puri M (2012). Immobilization of β-d-galactosidase from *Kluyveromyces lactis* on functionalized silicon dioxide nanoparticles: characterization and lactose hydrolysis. Int J Biol Macromol.

[26] Biró E, Budugan D, Todea A, Péter F, Klébert S, Feczkó T (2016). Recyclable solid-phase biocatalyst with improved stability by sol-gel entrapment of β-d-galactosidase. J Mol Catal B.

[27] Deressa T, Stöcklinger A, Wallner M, Himly M, Kofler S, Hainz K, Brandstetter H, Thalhamer J, Hammerl P (2014). Structural integrity of the antigen is a determinant for the induction of T-helper type-1 immunity in mice by gene gun vaccines against *E. coli* beta-galactosidase. PLoS ONE.

[28] Berger T, Sterrer M, Diwald O, Knözinger E, Panayotov D, Thompson TL, Yates JT (2005). Light-induced charge separation in anatase TiO_2_ particles. J Phys Chem B.

[29] Berger T, Sterrer M, Diwald O, Knözinger E (2005). Charge trapping and photoadsorption of O_2_ on dehydroxylated TiO_2_ nanocrystals-an electron paramagnetic resonance study. ChemPhysChem.

[30] Wallenfels K, Malhotra OP (1962). Galactosidases. Adv Carbohydr Chem.

[31] Barth A, Zscherp C (2002). What vibrations tell us about proteins. Q Rev Biophys.

[32] Barth A (2007). Infrared spectroscopy of proteins. Biochim Biophys Acta.

[33] Pettersen EF, Goddard TD, Huang CC, Couch GS, Greenblatt DM, Meng EC, Ferrin TE (2004). UCSF chimera-a visualization system for exploratory research and analysis. J Comput Chem.

[34] Márquez A, Berger T, Feinle A, Hüsing N, Himly M, Duschl A, Diwald O (2017). Langmuir.

[35] Moulton SE, Barisci JN, McQuillan AJ, Wallace GG (2003). ATR-IR spectroscopic studies of the influence of phosphate buffer on adsorption of immunoglobulin G to TiO_2_. Colloids Surf A.

[36] Wei T, Kaewtathip S, Shing K (2009). Buffer effect on protein adsorption at liquid/solid interface. J Phys Chem C.

[37] Mahmoudi M, Shokrgozar MA, Sardari S, Moghadam MK, Vali H, Laurent S, Stroeve P (2011). Irreversible changes in protein conformation due to interaction with superparamagnetic iron oxide nanoparticles. Nanoscale.

[38] Röcker C, Pötzl M, Zhang F, Parak WJ, Nienhaus GU (2009). A quantitative fluorescence study of protein monolayer formation on colloidal nanoparticles. Nat Nanotechnol.

[39] Casals E, Pfaller T, Duschl A, Oostingh GJ, Puntes V (2010). Time evolution of the nanoparticle protein corona. ACS Nano.

[40] Glassford SE, Byrne B, Kazarian SG (2013). Recent applications of ATR FTIR spectroscopy and imaging to proteins. Biochim Biophys Acta.

[41] Mudunkotuwa IA, Minshid AA, Grassian VH (2014). ATR-FTIR spectroscopy as a tool to probe surface adsorption on nanoparticles at the liquid-solid interface in environmentally and biologically relevant media. Analyst.

[42] Arrondo J, Muga A, Castresana J, Goñi FM (1993). Quantitative studies of the structure of proteins in solution by fourier-transform infrared spectroscopy. Prog Biophys Mol Biol.

[43] Arrondo J, Muga A, Castresana J, Bernabeu C, Goñi FM (1989). An infrared spectroscopic study of β-galactosidase structure in aqueous solutions. FEBS Lett.

[44] Juers DH, Matthews BW, Huber RE (2012). LacZ β-galactosidase: structure and function of an enzyme of historical and molecular biological importance. Protein Sci.

[45] Juers DH, Jacobson RH, Wigley D, Zhang XJ, Huber RE, Tronrud DE, Matthews BW (2000). High resolution refinement of β-galactosidase in a new crystal form reveals multiple metal-binding sites and provides a structural basis for α-complementation. Protein Sci.

[46] Cao L (2006). Carrier-bound immobilized enzymes: principles, application and design.

[47] Wehtje E, Adlercreutz P, Mattiasson B (1993). Improved activity retention of enzymes deposited on solid supports. Biotechnol Bioeng.

[48] Ni Y, Li J, Huang Z, He K, Zhuang J, Yang W (2013). Improved activity of immobilized horseradish peroxidase on gold nanoparticles in the presence of bovine serum albumin. J Nanopart Res.

