# Supplementary material for: Enzyme adsorption-induced activity changes: a quantitative study on TiO2 model agglomerates
Source: J Nanobiotechnology. 2017 Jul 21;15:55. doi: 10.1186/s12951-017-0283-4 (PMC5521066; doi:10.1186/s12951-017-0283-4)
Supplement: Supplementary file 1 — Additional file 1. Transmission electron micrographs, hydrolysis plots, IR spectra and fitting results. [file 12951_2017_283_MOESM1_ESM.docx]

Additional Supporting File

**Enzyme Adsorption-induced Activity Changes: A Quantitative Study on TiO_2_ Model Agglomerates**

Augusto Márquez,^a^ Krisztina Kocsis,^a^ Gregor Zickler,^a^ Gilles Bourret,^a^ Andrea Feinle,^a^ Nicola Hüsing,^a^ Martin Himly,^b,*^ Albert Duschl^b^, Thomas Berger,^a,*^ and Oliver Diwald^a^

^a^ *Department of Chemistry and Physics of Materials, Paris Lodron University of Salzburg, Jakob-Haringer-Strasse 2a, A - 5020 Salzburg, Austria*

^b^ *Department of Molecular Biology, Paris Lodron University of Salzburg, Hellbrunnerstrasse 34/III, A - 5020 Salzburg, Austria*

* Corresponding authors: [Thomas.Berger@sbg.ac.at](mailto:Thomas.Berger@sbg.ac.at)

[Martin.Himly@sbg.ac.at](mailto:Martin.Himly@sbg.ac.at)


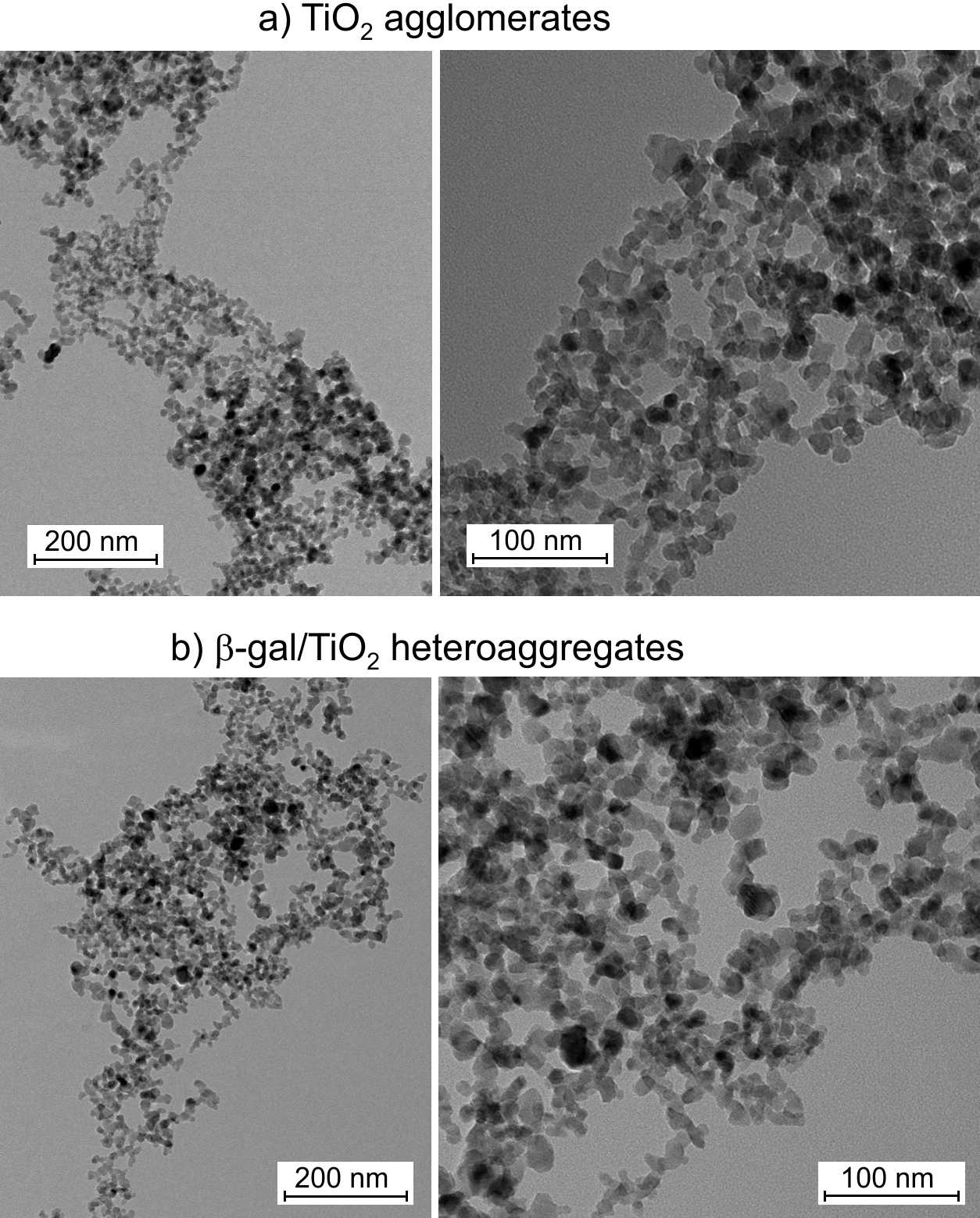


**Figure S1:** Transmission electron micrographs of (a) TiO_2_ agglomerates and (b) β-gal/TiO_2_ heteroaggregates.


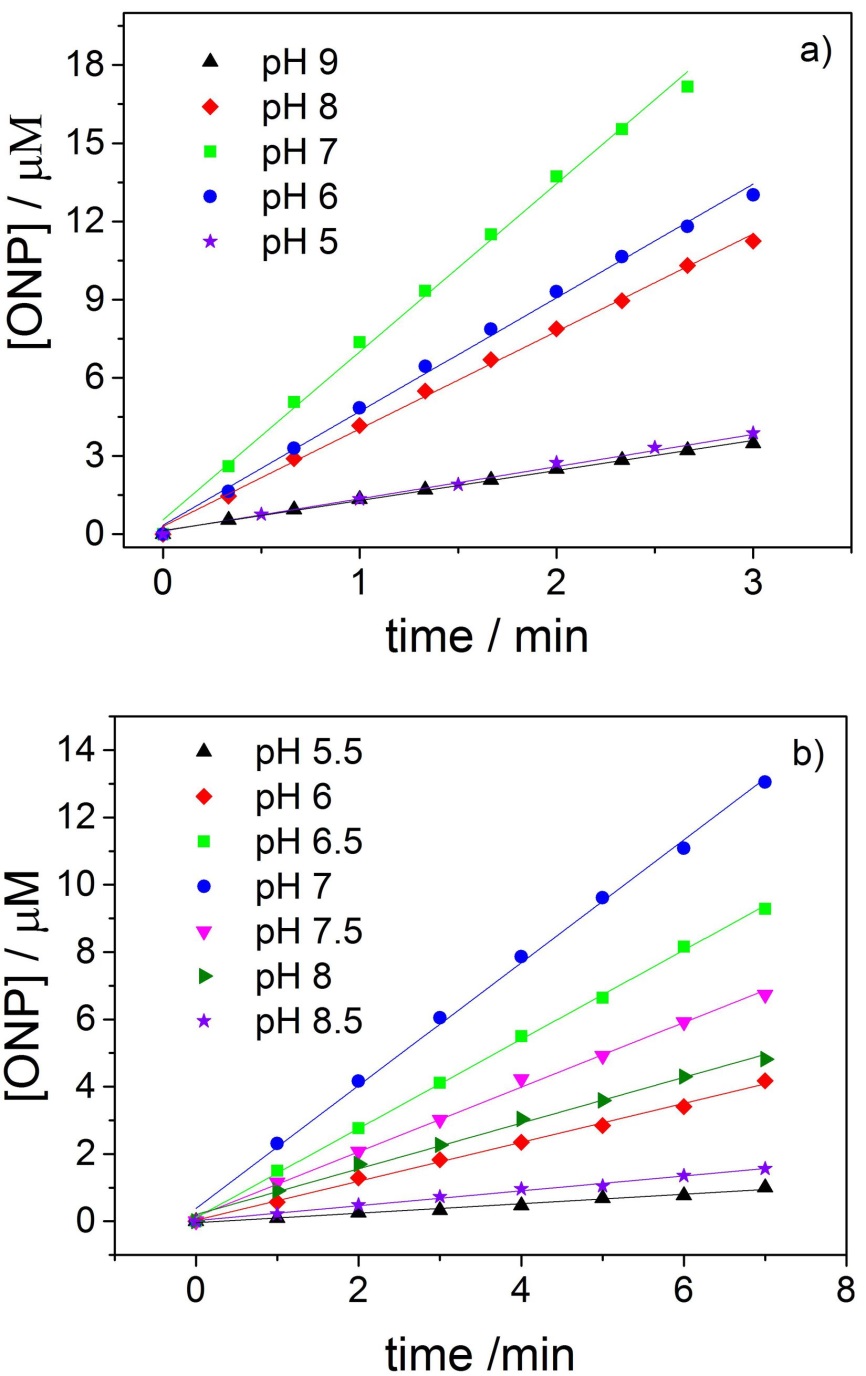


**Figure S2:** Hydrolysis plots for β-gal in McIlvaine´s buffer at 5 ≤ pH ≤ 9: a) free enzyme in solution ([β-gal] = 0.5 µg mL^-1^) and b) enzyme adsorbed on TiO_2_ nanoparticle agglomerates ([TiO_2_] = 1 mg mL^-1^, [β-gal] = 8.5 µg mL^-1^). MgCl_2_ (1 mM) and ONPG (0.5 mM) were used as cofactor and substrate, respectively. After protein adsorption and prior to the enzymatic assay β-gal/TiO_2_ heteroaggregates were washed first with water and then with the respective buffer solution. The reaction progress was sampled as a function of time by withdrawing 0.5 mL of the reaction mixture and mixing with 0.7 mL of 1 M Na_2_CO_3_ aqueous solution to stop the reaction. The concentration of the reaction product (o-Nitrophenol, ONP) was determined photometrically.


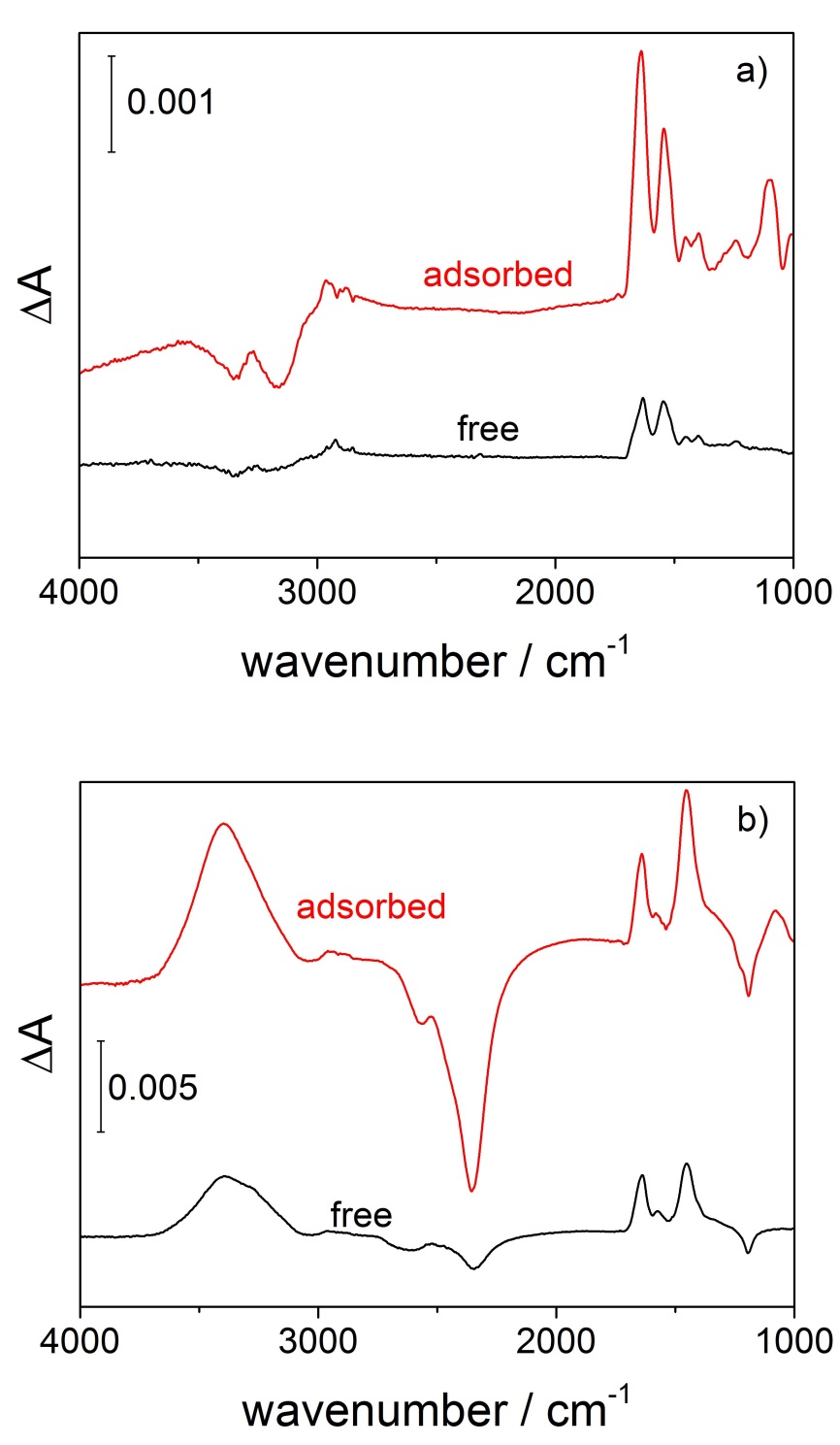


**Figure S3:** ATR-IR spectra of free β-gal in aqueous solution (([β-gal] = 150 µg mL^-1^; H_2_O (a) and D_2_O (b)) and of β-gal adsorbed on porous films of TiO_2_ nanoparticles ([β-gal] = 150 µg mL^-1^, [TiO_2_] = 1.3 mg mL^-1^, adsorption time: 8 h), background spectra: protein-free aqueous solution (H_2_O (a) and D_2_O (b)) in contact with the uncovered ATR prism or with the TiO_2_ film, respectively.


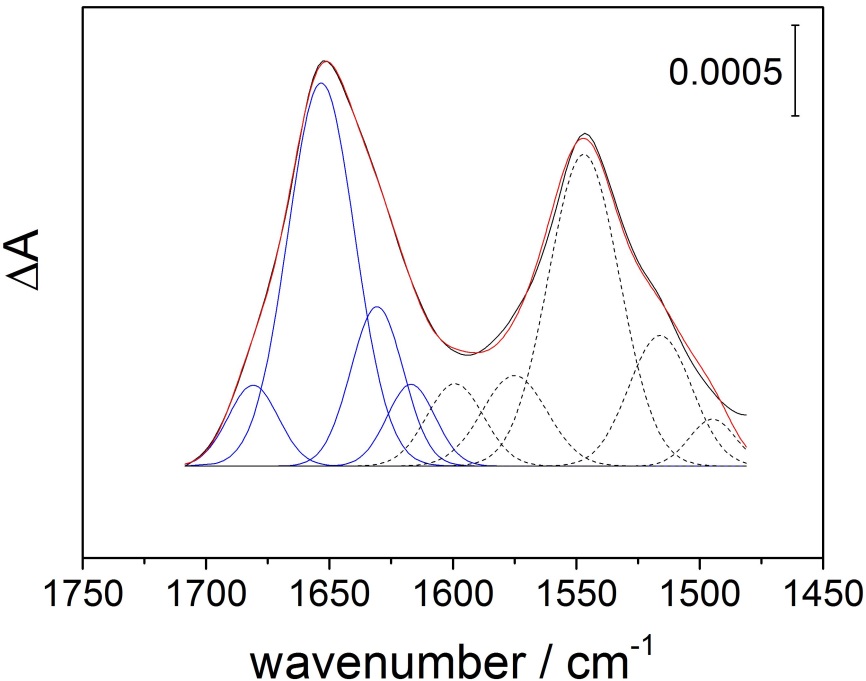


**Figure S4:** Fitting result for β-gal adsorbed on a porous film of TiO_2_ nanoparticles ([β-gal] = 150 µg mL^-1^, aqueous (H_2_O) solution, [TiO_2_] = 1.3 mg mL^-1^, adsorption time: 8 h), background spectrum: protein-free aqueous solution (H_2_O) in contact with the TiO_2_ film.


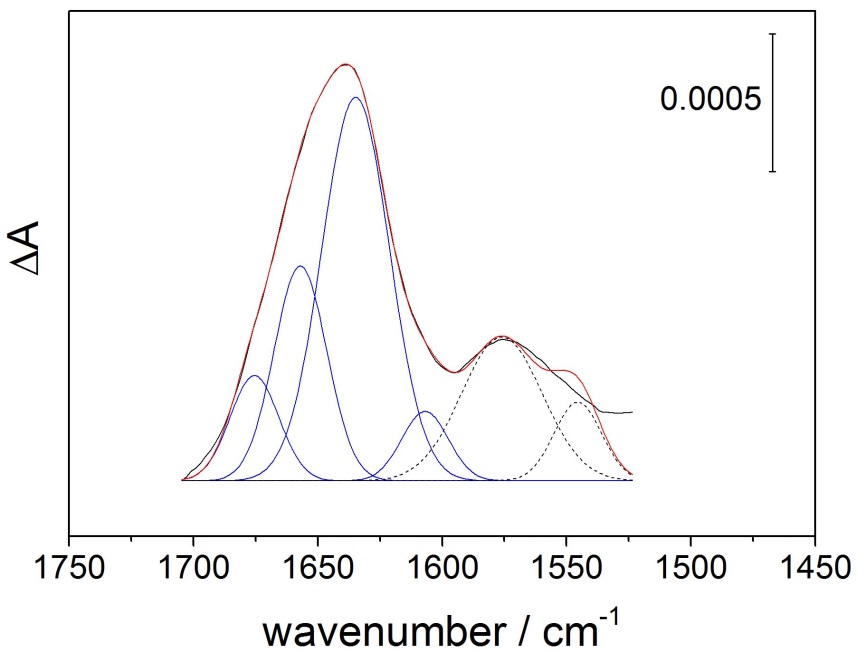


**Figure S5:** Fitting result for β-gal adsorbed on a porous film of TiO_2_ nanoparticles ([β-gal] = 150 µg mL^-1^, aqueous (D_2_O) solution, [TiO_2_] = 1.3 mg mL^-1^, adsorption time: 8 h), background spectrum: protein-free aqueous solution (D_2_O) in contact with the TiO_2_ film.

**
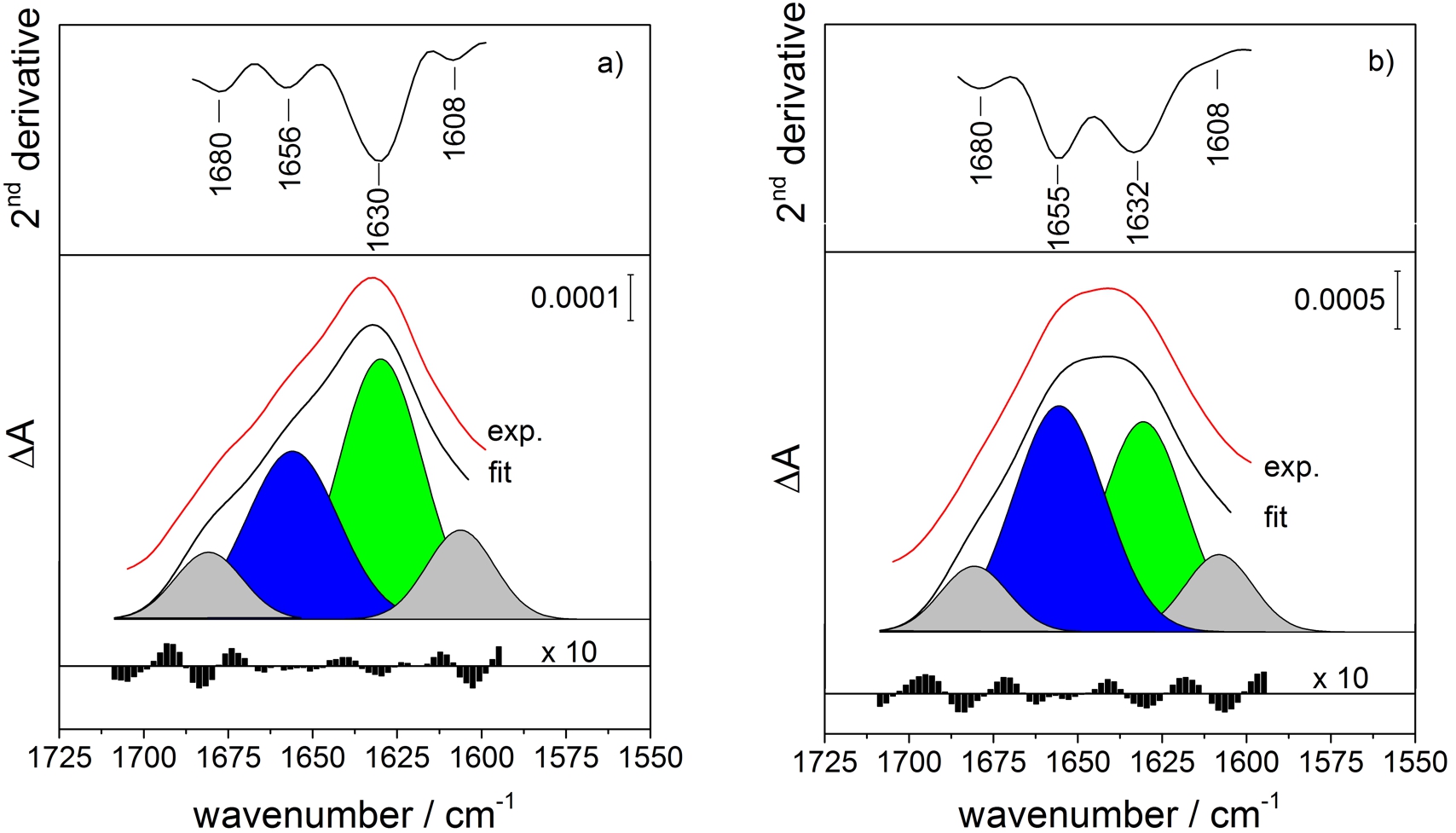
**

**Figure S6:** Second derivative of the amide I band, fitting results and corresponding residuals for (a) free β-gal in H_2_O ([β-gal] = 150 µg mL^-1^) and (b) β-gal adsorbed on a porous film of TiO_2_ nanoparticles ([β-gal] = 150 µg mL^-1^, [TiO_2_] = 1.3 mg mL^-1^, adsorption time: 8 h), background spectrum: TiO_2_ film in contact with H_2_O. The band parameters of the deconvoluted single components are listed in Table S1.

**Table S1:** Band parameters of deconvoluted single components contributing to the amide I band of free β-gal in H_2_O and β-gal adsorbed on TiO_2_ (corresponding to the fitting results represented in Figure S8).

| **Structure** | **Free β-gal** | | | **Adsorbed β-gal** | | |
| --- | --- | --- | --- | --- | --- | --- |
|  | Peak position / cm^-1^ | FWHM / cm^-1^ | Area /  % | Peak position / cm^-1^ | FWHM / cm^-1^ | Area /  % |
| Inter β-sheet | 1608 | 24 | 13 | 1608 | 24 | 11 |
| β-sheet | 1630 | 29 | 46 | 1632 | 29 | 37 |
| α-helix + random | 1656 | 32 | 32 | 1655 | 32 | 43 |
| turn | 1680 | 24 | 9 | 1680 | 24 | 9 |
